# Intrasaccular flow disruption (WEB) of a large wide-necked basilar apex aneurysm using PulseRider-assistance

**DOI:** 10.1016/j.inat.2020.101072

**Published:** 2020-12-29

**Authors:** Kazim H. Narsinh, M. Travis Caton, Nausheen F. Mahmood, Randall T. Higashida, Van V. Halbach, Steven W. Hetts, Matthew R. Amans, Christopher F. Dowd, Daniel L. Cooke

**Affiliations:** a Department of Radiology & Biomedical Imaging, University of California San Francisco, San Francisco, CA 94143, USA; b Department of Neurology, University of California San Francisco, San Francisco, CA 94143, USA

**Keywords:** Basilar apex aneurysm, Wide-necked, Intrasaccular flow disruption

## Abstract

Large, wide-necked basilar apex aneurysms are difficult to treat. Microsurgical clipping can result in neurologic morbidity and mortality. Endovascular treatment often leaves remnants that need retreatment and/or stent placement with dual antiplatelet therapy. The Woven EndoBridge (WEB) is an intrasaccular flow disruption device that can be used without dual antiplatelet therapy. However, the WEB cannot typically be used in large or giant aneurysms *>* 10 mm because the largest diameter device is the 11 × 9.6 mm single layer sphere (SLS). We present a case in which we use a PulseRider aneurysm neck reconstruction device in the basilar artery to assist in WEB deployment within a 22 mm basilar apex aneurysm with 14 mm neck, thereby permitting aspirin monotherapy postoperatively.

## 1. Introduction

Operative complications of microsurgical clipping for basilar apex aneurysms such as cranial nerve or perforator injury can result in neurologic deficit or death in up to 10–50% of patients [1–3]. While endovascular treatment often decreases perioperative morbidity and mortality, aneurysm remnants and retreatment are common [4]. Better results of endovascular surgery can be achieved using stents [5,6], although this necessitates use of dual antiplatelet therapy, which is not without its own risks. Furthermore, good outcomes are especially difficult to achieve in giant or large, wide-necked basilar apex aneurysms. Herein we present a case using a WEB to treat a large, wide-necked basilar apex aneurysm in conjunction with a PulseRider neck reconstruction device.

## 2. Case report

**Fig. 1.**
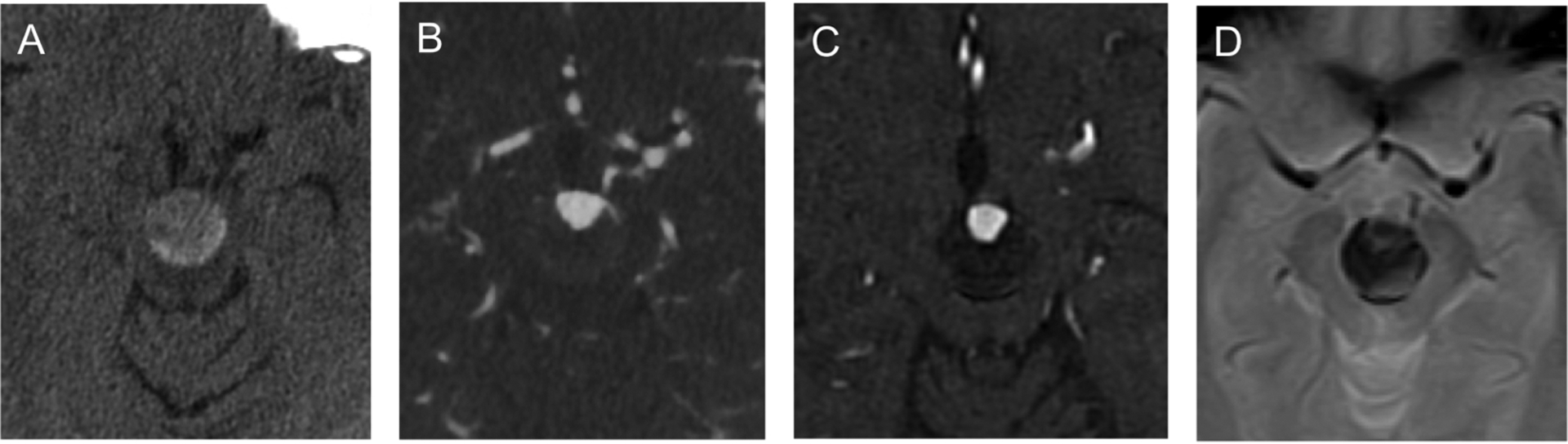
(A) Axial unenhanced CT demonstrates basilar apex aneurysm measuring 20 mm in width in the interpeduncular fossa. (B) Axial CTA shows aneurysmal lumen measuring 12 mm in width at its base. (C) Time-of-flight MRA in axial plane shows the aneurysmal lumen in relationship to the partially thrombosed aneurysm. (D) Axial GRE shows partially thrombosed basilar apex aneurysm and no subarachnoid hemorrhage.

A woman in her 7th decade of life with history of hypertension, tobacco use, and methamphetamine use presented with acute-onset, severe headache, neck pain, and intermittent diplopia to another institution. She had no focal neurologic deficits on exam. No subarachnoid hemorrhage was seen on head CT or MRI, and she did not undergo a lumbar puncture. CTA and MRA showed a 20 mm, partially-thrombosed, wide-necked basilar apex aneurysm (Fig. 1).

**Fig. 2.**
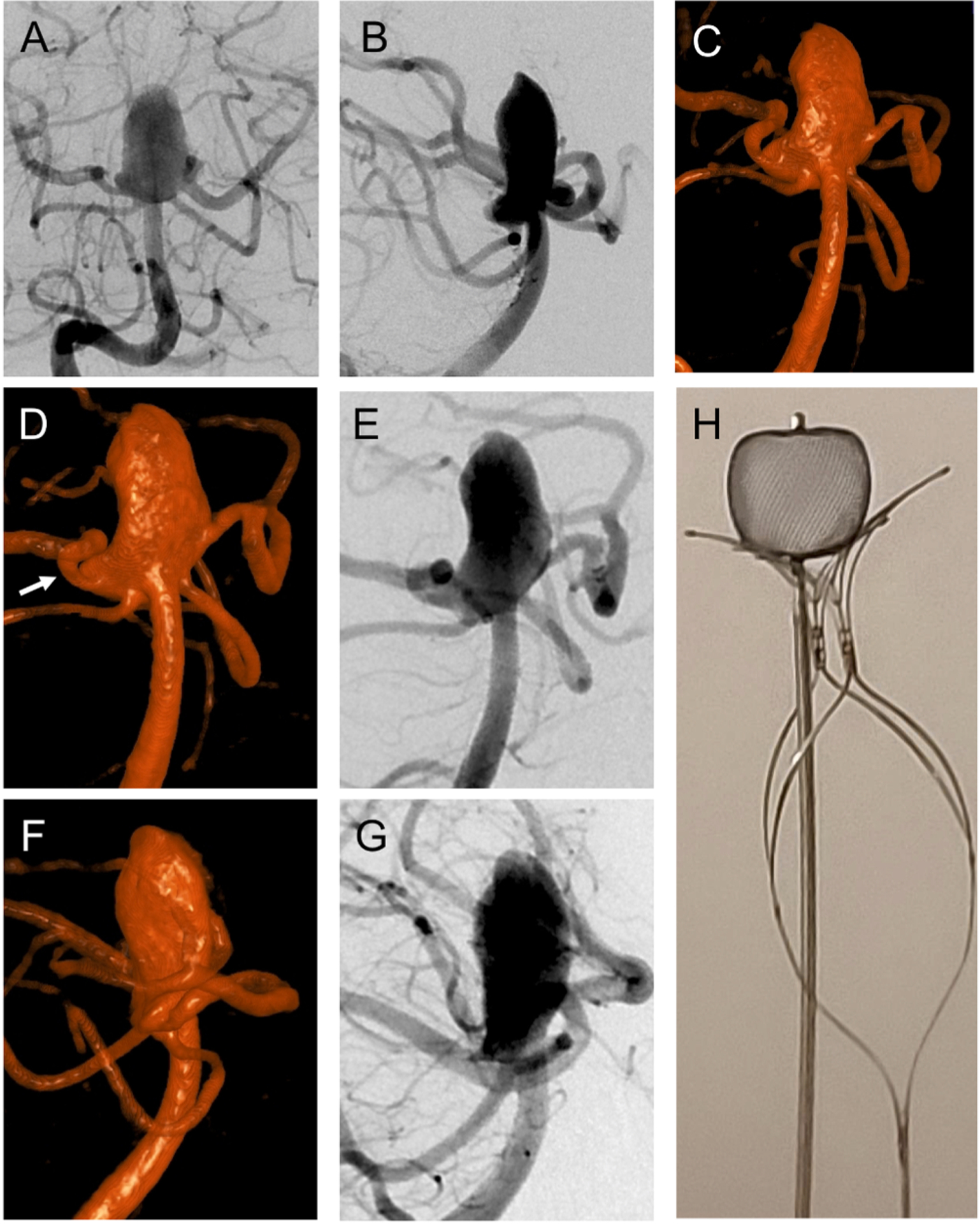
(A) Right vertebral arteriogram in frontal projection shows wide-necked, large basilar apex aneurysm incorporating both PCA origins. (B) Lateral projection of right vertebral arteriogram shows the relationship of both PCAs and SCAs to the aneurysm. (C) Volume-rendered reformat of the flat panel CTA showing the extension of the aneurysm into the right PCA origin. (D) Volume-rendered reformat of the flat panel CTA in the right anterior oblique Water’s projection for treatment. (E) Right anterior oblique Water’s projection (same as D) of right vertebral arteriogram for treatment. (F) Volume-rendered reformat of the flat panel CTA in the right posterior oblique Schuller’s projection for treatment. (G) Right posterior oblique Schuller’s projection (same as F) of right vertebral arteriogram for treatment. (H) Ex vivo demonstration of WEB SLS 11 × 9.6 mm atop PulseRider Y-shape with 10.6 mm arch width.

Cerebral angiography demonstrated a 20 × 19 × 18 mm (height × width × length) partially-thrombosed basilar apex aneurysm, with aneurysmal lumen measuring 18 × 10 × 7 mm (height × width × length), and a 12 mm neck incorporating both posterior cerebral artery (PCA) origins (Fig. 2). At another institution, using a dual-microcatheter setup, a 12 mm coil was deployed into the aneurysm but not detached, and a microwire was advanced into the right PCA. However, the microcatheter could not be advanced over the microwire into the right PCA. During attempted removal of the coil, it broke, then was retrieved using a 4 mm snare. The aneurysm was left unsecured and the patient was transferred to our institution.

**Fig. 3.**
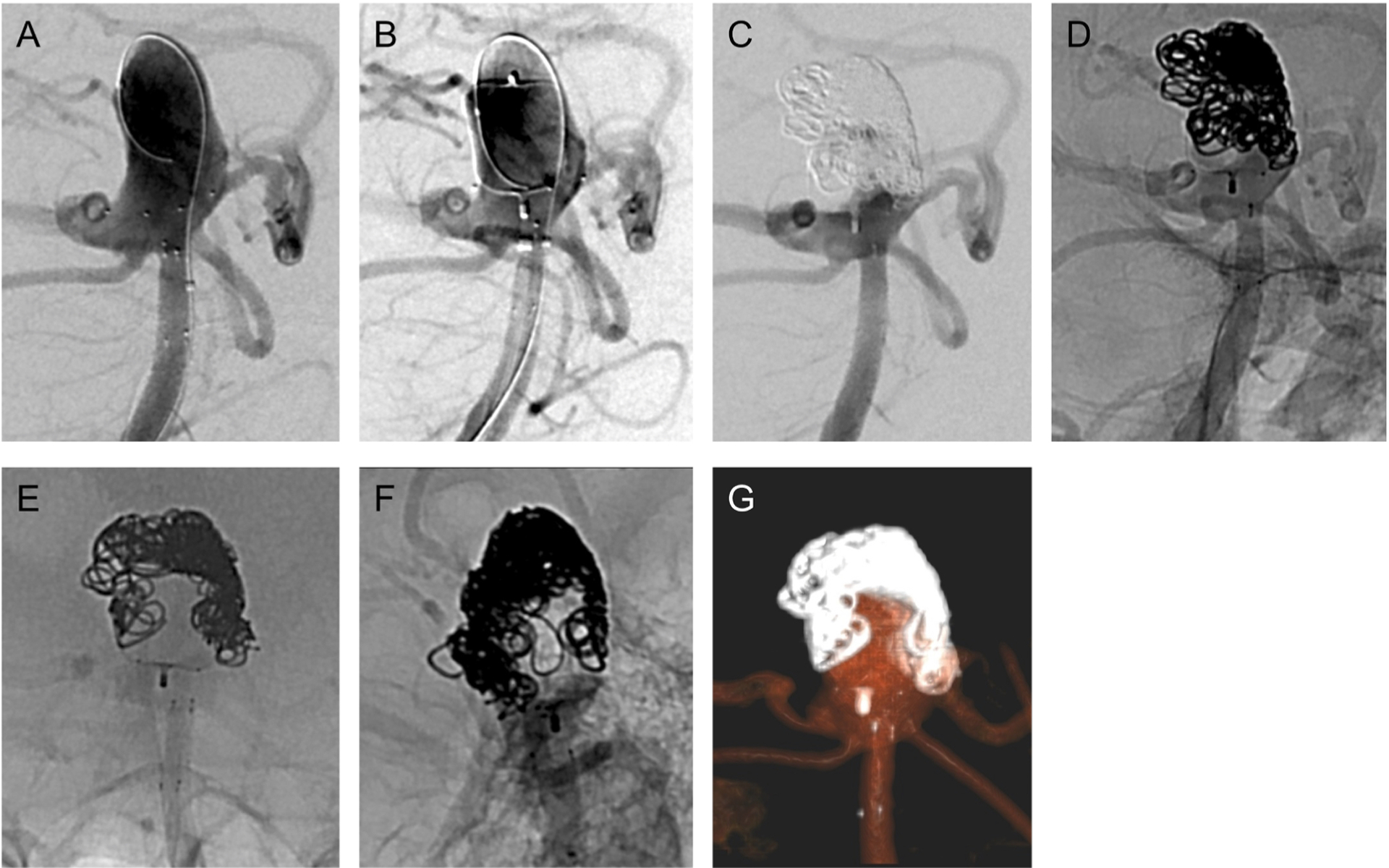
(A) Vertebral arteriogram in same projection as Fig. 2D–E shows the coiling catheter looped at the dome of the aneurysm lumen and the PulseRider deployed in the distal basilar artery. (B) Vertebral arteriogram in same projection after deploying and detaching WEB atop the PulseRider. The left aneurysm base is not covered by the WEB, and contrast reaches the aneurysm lumen’s dome after passing through the left aneurysm neck. (C) Vertebral arteriogram in same projection after placing multiple platinum and gel coils. The coils were placed atop the WEB and at the left aneurysm base. The aneurysm lumen’s dome is protected. (D) Unsubtracted fluoroscopic image (in same projection as Fig. 3C) demonstrating position of coils. (E) Unsubtracted fluoroscopic image (in same projection as Fig. 2A) showing coils atop the WEB atop the PulseRider. (F) Unsubtracted fluoroscopic image (in same projection as Fig. 2B) showing the same. (G) Volume-rendered reformat of dual-volume flat panel CTA showing the WEB, PulseRider, and coil construct.

Via right radial and left common femoral arterial accesses, we gained bilateral vertebral artery access. Via the left vertebral artery intermediate catheter, we looped a coiling catheter (Excelsior SL-10; Stryker) in the basilar apex aneurysm (Fig. 3). Then, via the right vertebral artery guide catheter, we advanced a microcatheter (Prowler Select Plus; Codman Johnson & Johnson) into the distal basilar artery, and deployed a PulseRider aneurysm neck reconstruction device (T-shape, 10 mm, 2.7–3.5 mm; Cerenovus Johnson & Johnson) with arch in the aneurysm base and anchor in the distal basilar artery (Fig. 3). Then, via the right vertebral artery guide catheter, we advanced a Via 33 microcatheter (Sequent Microvention) over a Headway Duo 156 (Microvention Terumo) microcatheter over a Synchro-2 microwire (Stryker Neurovascular) through the central lumen of the PulseRider into the aneurysm, taking care not to dislodge the PulseRider. Through the Via 33 microcatheter, we then deployed a WEB SL 10 × 7 mm device within the aneurysm lumen, seating it atop the arch of the PulseRider (Fig. 3). Then, via the coiling catheter already jailed in the aneurysm lumen, we placed multiple detachable coils (Target; Stryker and Hydrocoil; Microvention) with secondary diameters ranging from 7 mm to 3 mm at the superior and left aspect of the WEB.

Aneurysmal dilation of the origin of the right PCA persists, but the remaining aneurysm was occluded at case conclusion. No clinically significant thromboembolic complications occurred during the procedure. We caused a minor iatrogenic dissection of the proximal left vertebral artery during the procedure, which was not flow-limiting and has required no further treatment. The patient continued taking aspirin 81 mg daily as antiplatelet monotherapy after being loaded with 650 mg of aspirin orally on the day of the surgery. After the procedure, the patient stopped wearing her eyepatch, remained neurologically intact on examination, and was counseled to cease tobacco and methamphetamine use.

## 3. Discussion

Large, wide-necked basilar apex aneurysms are difficult to treat [7] because they have higher rates of operative complication [1–3] and need for retreatment [4–6]. Endovascular retreatment rates of wide-necked aneurysms have been reduced by use of self-expanding stents to reconstruct the aneurysm neck [8]. In a *meta*-analysis of 601 wide-necked bifurcation aneurysms treated with Y-stent-assisted coiling, of which 39% were located at the basilar tip, thromboembolism was the most common complication, occurring in 6.7% of cases, while acute stent occlusion was reported in 2% of cases [9]. In a series of 235 patients with basilar apex aneurysms treated with and without stents at a single institution, the rate of thromboembolism was 6.8% in both groups [8]. However, for very large (≥ 2 cm) or giant (≥ 2.5 cm) ruptured or unruptured intracranial aneurysms treated with endovascular reconstruction, ischemic complication rates rise considerably to 15–19%, with an overall complication rate of 30–34% [10]. In addition, stent-assisted coil embolization typically requires administration of dual antiplatelet therapy for 3–6 months, which is associated with an increased rate of major and minor intracranial and extracranial bleeding events [11].

The WEB device has emerged as a novel instrasaccular flow disruptor that can be used to treat wide-necked bifurcation aneurysms without dual antiplatelet therapy in select cases [12,13]. For example, only 11% of patients in the WEB-IT trial were taking dual antiplatelet therapy at the 6 month follow-up visit [12]. However, large aneurysms with width *>* 10 mm cannot typically be treated using WEB because the largest device is a 11 × 9.6 mm SLS, and adequate lateral wall-apposition is critical to ensure flow disruption at the aneurysm neck. These challenging aneurysms are not represented in published series using WEB because of the device dimensions. For the smaller aneurysms included in these trials, an adjunctive device, namely a flow-diverter or stent, was used in only 1.9% of patients treated with the single-layer WEB [14], but retreatment rates over 3 years can approach 11% [13].

A variety of advances in endovascular technology have enabled more durable treatment of wide-necked bifurcation aneurysms by reconstructing the aneurysm neck, including self-expanding stents in Y-, T-, or waffle cone configuration, the pCONus or pCANvas (Phenox), eClips (Evasc), or PulseRider (Cerenovus). Self-expanding stents in Y- or T- configuration or the eClips device necessitate catheterization of the branch vessels for deployment. In a case series of 24 patients treated with the eCLIPs device, one patient (4.6%) died due to guidewire perforation [15]. The durability and safety of endovascular treatment for large or giant wide-necked bifurcation aneurysms could be improved by eliminating the need to catheterize multiple branch vessels such as both posterior cerebral arteries, which may be considered advantages of the PulseRider, pCONus, and pCANvas devices. In addition, intrasaccular flow-disruption at the aneurysm neck using WEB may simplify treatment by reducing the number of steps to the procedure. However, no prior studies have described use of the WEB in conjunction with the newer neck reconstruction devices.

Use of the PulseRider or pCONUS for aneurysm neck reconstruction offers two potential advantages over cylindrical self-expanding stents (e.g. Y- or T-Stenting). First, both devices can be deployed without catheterizing branch vessels. Catheterizing branch vessels such as the posterior cerebral arteries can increase risk of intraoperative complications such as perforation or thromboembolism. Second, a lack of dual antiplatelet therapy is better tolerated because of the low metal coverage of these devices relative to braided (e.g. LVIS) or laser-cut (e.g. Neuroform Atlas) self-expanding stents. Although dual antiplatelet therapy is recommended by the manufacturer of PulseRider, prolonged dual antiplatelet therapy increases risk of intracranial and extracranial hemorrhage compared to single agent antiplatelet therapy [11]. Limited case reports and series have reported use of PulseRider or pCONus HPC in the setting of subarachnoid hemorrhage with the hypothesis that dual antiplatelet therapy may be deferred based on the low metal coverage of these devices [16–18], although long-term data in larger series is not yet available. Newer devices including the Contour and NeqStent neck bridging device (Cerus) may provide additional options for treating these challenging aneurysms endovascularly, although long-term follow-up data is needed.

The pCONus is a laser-cut, electrolytically-detachable, resheathable, self-expanding stent-like device with a distal crown and four petals that deploy within the aneurysm and bridge the aneurysm neck. A *meta*-analysis of 200 wide-necked bifurcation aneurysms treated with pCONus was notable for a thromboembolic complication rate of 12.1% [19]. pCONus has been used in a small series of 21 patients with acutely ruptured aneurysms, along with dual antiplatelet therapy, resulting in a 62% rate of modified Raymond-Roy classification 1–2 at immediate angiography [20]. The pCANvas is a newer derivate of the pCONus with similar structure and delivery but with the added feature of a membrane between each petal, designed to further augment the neck-bridging effect [21,22]. The pCONus and pCANvas are not yet available in the United States. The PulseRider is a self-expanding nitinol stent with 4.5% metal surface coverage that can be placed intrasaccular or intraluminal to reconstruct wide aneurysm necks and buttress an intrasaccular coil mass, preventing coil herniation into the parent artery. The PulseRider facilitates adequate occlusion of ~ 90% of wide-necked bifurcation aneurysms on immediate postoperative angiography [23]. A treatment approach that avoids catheterizing branch vessels or using dual antiplatelet therapy could improve the safety of endovascular treatment for large, wide-necked bifurcation aneurysms. Although PulseRider is typically used in conjunction with coils, use of multiple coils in a partially thrombosed aneurysm can increase risk of thromboembolic complication if adequate neck coverage is desired. Herein, we describe use of the WEB in conjunction with a PulseRider below and coils above to treat a large, wide-necked basilar apex aneurysm. ThePulseRider, anchored in the basilar artery, effectively buttressed the WEB to prevent its downward herniation into the parent vessel, while coils deployed above the WEB mitigate upward displacement and compression of the WEB into the aneurysm, a factor which accounts for 26.6% of post-WEB retreatments in one series [24]. The principle advantage of this approach is that both devices can be deployed perpendicular to the aneurysm neck, obviating the need for daughter vessel catheterization which can pose considerable difficulty when these vessels are dysplastic or tortuous, thereby overcoming a major technical limitation of stent- or balloon-assisted WEB deployment.

Several limitations of this technique merit additional discussion. First, although initial angiographic results were excellent, the durability of this treatment is not certain. Should interval growth of the dysplastic P1 PCA segments occur, retreatment may be needed using cylindrical self-expanding stent. Next, when using the above-described approach, care must be taken to not dislodge the PulseRider when crossing its central lumen to place the WEB. This can be facilitated by using a smaller microcatheter (e.g. Headway 156) within the Via 33 microcatheter to reduce the ledge effect. Jailing a microcatheter in the aneurysm can also be helpful for adjunctive coiling.

In summary, the combination use of the WEB, PulseRider, and coils does not require catheterization of branch vessels or use of dual antiplatelet therapy, which may improve safety, particularly in the treatment of ruptured, large, wide-necked bifurcation aneurysms. This technique leverages the complimentary advantages of each device, expanding the scope of aneurysms amenable to endovascular surgery.

## References

[1] Lozier AP, Kim GH, Sciacca RR, Connolly ES, Solomon RA, Microsurgical Treatment of Basilar Apex Aneurysms: Perioperative and Long-term Clinical Outcome, Neurosurgery. 54 (2) (2004) 286–299. 14744274 10.1227/01.neu.0000103222.13642.00

[2] Lawton MT, Basilar Apex Aneurysms: Surgical Results and Perspectives from an Initial Experience, Neurosurgery. 50 (1) (2002) 1–10. 11844228 10.1097/00006123-200201000-00002

[3] Tjahjadi M, Serrone J, Hernesniemi J, Should we still consider clips for basilar apex aneurysms? A critical appraisal of the literature, Surg Neurol Int. 21 (2018) 9. 10.4103/sni.sni_311_17 PMC5843972 29541485

[4] Henkes H, Fischer S, Mariushi W, Weber W, Liebig T, Miloslavski E, , Angiographic and clinical results in 316 coil-treated basilar artery bifurcation aneurysms, J Neurosurg. 103 (6) (2005) 990–999. 16381185 10.3171/jns.2005.103.6.0990

[5] Tjahjadi M, Kim T, Ojar D, Byoun HS, Lee SU, Ban SP, , Long-term review of selected basilar-tip aneurysm endovascular techniques in a single institution, Interdisciplinary Neurosurgery. 1 (8) (2017) 50–56.

[6] Phan K, Huo YR, Jia F, Phan S, Rao PJ, Mobbs RJ, , Meta-analysis of stent-assisted coiling versus coiling-only for the treatment of intracranial aneurysms, J Clin Neurosci. 31 (2016) 15–22. 27344091 10.1016/j.jocn.2016.01.035

[7] Fiorella D, Arthur AS, Chiacchierini R, Emery E, Molyneux A, Pierot L, How safe and effective are existing treatments for wide-necked bifurcation aneurysms? Literature-based objective performance criteria for safety and effectiveness, J Neurointerv Surg. 9 (12) (2017) 1197–1201. 28798268 10.1136/neurintsurg-2017-013223

[8] Chalouhi N, Jabbour P, Gonzalez LF, Dumont AS, Rosenwasser R, Starke RM, , Safety and efficacy of endovascular treatment of basilar tip aneurysms by coiling with and without stent assistance: a review of 235 cases, Neurosurgery. 71 (4) (2012) 785–794. 22743359 10.1227/NEU.0b013e318265a416

[9] Cagnazzo F, Limbucci N, Nappini S, Renieri L, Rosi A, Laiso A, , Y-Stent-Assisted Coiling of Wide-Neck Bifurcation Intracranial Aneurysms: A Meta-Analysis, AJNR Am J Neuroradiol. 40 (1) (2019) 122–128. 30523146 10.3174/ajnr.A5900 PMC7048580

[10] Cagnazzo F, Mantilla D, Rouchaud A, Brinjikji W, Lefevre P-H, Dargazanli C, , Endovascular Treatment of Very Large and Giant Intracranial Aneurysms: Comparison between Reconstructive and Deconstructive Techniques-A Meta-Analysis, AJNR Am J Neuroradiol. 39 (5) (2018) 852–858. 29545248 10.3174/ajnr.A5591 PMC7410657

[11] Naqvi IA, Kamal AK, Rehman H, Multiple versus fewer antiplatelet agents for preventing early recurrence after ischaemic stroke or transient ischaemic attack, Cochrane Database Syst Rev. 8 (2020). CD009716. 32813275 10.1002/14651858.CD009716.pub2 PMC7437397

[12] Arthur AS, Molyneux A, Coon AL, Saatci I, Szikora I, Baltacioglu F, , The safety and effectiveness of the Woven EndoBridge (WEB) system for the treatment of wide-necked bifurcation aneurysms: final 12-month results of the pivotal WEB Intrasaccular Therapy (WEB-IT) Study, J Neurointerv Surg. 11 (9) (2019 9) 924–930. 30992395 10.1136/neurintsurg-2019-014815 PMC6824604

[13] Pierot L, Szikora I, Barreau X, Holtmannspoetter M, Spelle L, Herbreteau D, , Aneurysm treatment with WEB in the cumulative population of two prospective, multicenter series: 3-year follow-up, J Neurointerv Surg. (2020) 12. 10.1136/neurintsurg-2020-016151 PMC7982938 32532858

[14] Pierot L, Gubucz I, Buhk JH, Holtmannspötter M, Herbreteau D, Stockx L, , Safety and Efficacy of Aneurysm Treatment with the WEB: Results of the WEBCAST 2 Study, American Journal of Neuroradiology. 38 (6) (2017) 1151–1155. 28450432 10.3174/ajnr.A5178 PMC7960101

[15] De Vries J, Boogaarts HD, Sørensen L, Holtmannspoetter M, Benndorf G, Turowski B, , eCLIPs bifurcation remodeling system for treatment of wide neck bifurcation aneurysms with extremely low dome-to-neck and aspect ratios: a multicenter experience, J Neurointerv Surg. (2020). 10.1136/neurintsurg-2020-016354 PMC8053345 32788388

[16] O’Connor KP, Strickland AE, Bohnstedt BN, PulseRider Use in Ruptured Basilar Apex Aneurysms, World Neurosurg. 127 (2019) 346–349. 30980983 10.1016/j.wneu.2019.04.042

[17] Folzenlogen Z, Seinfeld J, Kubes S, Kumpe D, Case D, Roark C, Use of the PulseRider Device in the Treatment of Ruptured Intracranial Aneurysms: A Case Series, World Neurosurg. 127 (2019) e149–e154. 30862588 10.1016/j.wneu.2019.03.003

[18] Aguilar Perez M, AlMatter M, Hellstern V, Wendl C, Ganslandt O, Bäzner H, , Use of the pCONus HPC as an adjunct to coil occlusion of acutely ruptured aneurysms: early clinical experience using single antiplatelet therapy, J Neurointerv Surg. 12 (9) (2020) 862–868. 32102920 10.1136/neurintsurg-2019-015746 PMC7476363

[19] Krupa K, Brzegowy P, Kucybała I, Łasocha B, Urbanik A, Popiela TJ, Endovascular embolization of wide-necked bifurcation aneurysms with the use of pCONus device: A systematic review and meta-analysis, Clin Imaging. 24 (70) (2020) 81–88. 10.1016/j.clinimag.2020.10.025 33130244

[20] Pérez MA, Bhogal P, Moreno RM, Wendl C, Bäzner H, Ganslandt O, , Use of the pCONus as an adjunct to coil embolization of acutely ruptured aneurysms, J Neurointerv Surg. 9 (1) (2017) 39–44. 27411859 10.1136/neurintsurg-2016-012508 PMC5264233

[21] Lylyk P, Chudyk J, Bleise C, Henkes H, Bhogal P, Treatment of Wide-Necked Bifurcation Aneurysms : Initial Results with the pCANvas Neck Bridging Device, Clin Neuroradiol. 29 (3) (2019) 467–477. 29556668 10.1007/s00062-018-0680-6 PMC6710216

[22] Sirakov S, Panayotova A, Sirakov A, Penkov M, Minkin K, Hristov H, Using the pCANvas neck-bridging device in treating a wide-necked aneurysm of the basilar tip, Neuroradiol J. 32 (3) (2019) 193–199. 30942655 10.1177/1971400919839375 PMC6512211

[23] Pranata R, Yonas E, Vania R, Sidipratomo P, July J, Efficacy and safety of PulseRider for treatment of wide-necked intracranial aneurysm-A systematic review and meta-analysis, Interv Neuroradiol. (2020). 10.1177/1591019920940521 PMC7903548 32635777

[24] Kabbasch C, Goertz L, Siebert E, Herzberg M, Hamisch C, Mpotsaris A, , Treatment strategies for recurrent and residual aneurysms after Woven Endobridge implantation, Journal of NeuroInterventional Surgery. 11 (4) (2019) 390–395. 30154251 10.1136/neurintsurg-2018-014230

